# Exploring the Potential Enhancing Effects of Trans-Zeatin and Silymarin on the Productivity and Antioxidant Defense Capacity of Cadmium-Stressed Wheat

**DOI:** 10.3390/biology11081173

**Published:** 2022-08-04

**Authors:** Esmat F. Ali, Alshafei M. Aljarani, Fozia A. Mohammed, El-Sayed M. Desoky, Ibrahim A. A. Mohamed, Mohamed El-Sharnouby, Suzan A. Tammam, Fahmy A. S. Hassan, Mostafa M. Rady, Ahmed Shaaban

**Affiliations:** 1 Department of Biology, College of Science, Taif University, P.O. Box 11099, Taif 21944, Saudi Arabia; 2 Botany Department, Faculty of Science, University of Sebha, Sebha 18758, Libya; 3 Botany Department, Faculty of Agriculture, Zagazig University, Zagazig 44519, Egypt; 4 Botany Department, Faculty of Agriculture, Fayoum University, Fayoum 63514, Egypt; 5 Department of Biotechnology, College of Science, Taif University, P.O. Box 11099, Taif 21944, Saudi Arabia; 6 Department of Botany and Microbiology, Faculty of Science, Assiut Unversity, Assiut 71515, Egypt; 7 Biology Department, Faculty of Science, Al-Baha University, Al-Baha 65779-77388, Saudi Arabia; 8 Horticulture Department, Faculty of Agriculture, Tanta University, Tanta 31527, Egypt; 9 Agronomy Department, Faculty of Agriculture, Fayoum University, Fayoum 63514, Egypt

**Keywords:** cadmium toxicity, *Triticum aestivum* L., growth and grain yield, gene expression, antioxidant defense systems, phytohormones, silymarin

## Abstract

**Simple Summary:**

Wheat experiments have provided insight into tolerance to cadmium (Cd) stress, the way in which wheat alters its morpho-physio-biochemical and antioxidant system responses when *trans*-Zeatin + silymarin (applied as seed priming + leaf spray) treatment is offered against Cd stress. This integrative treatment effectively enhanced growth, productivity, photosynthetic efficiency, leaf integrity, and antioxidant systems in the Cd-stressed wheat plants. This treatment reduced the Cd contamination (healthy grains) and increased growth and productivity by increasing osmo-regulatory compounds along with different antioxidant activities, which serve as potent defenses to protect plants from Cd stress by increasing tolerance to Cd stress in wheat.

**Abstract:**

Pot trials were performed to explore the impacts of seed priming (SPr) plus leaf treatment (LTr) with *trans*-zeatin-type cytokinin (*t*Zck; 0.05 mM) and silymarin (Sim; 0.5 mM) on growth, yield, physio-biochemical responses, and antioxidant defense systems in Cd-stressed wheat. *t*Zck + Sim applied as SPr + LTr was more effective than individual treatments, and the impacts were more pronounced under stress conditions. Cd stress (0.6 mM) severely declined growth and yield traits, and photosynthesis efficiency (pigment contents, instantaneous carboxylation efficiency, and photochemical activity) compared to the control. These negative impacts coincided with increased levels of Cd^2+^, O_2_^•−^ (superoxide), H_2_O_2_ (hydrogen peroxide), MDA (malondialdehyde), and EL (electrolyte leakage). Non-enzymatic and enzymatic antioxidant activities, and *t*Zck and Sim contents were also increased. However, tZck + Sim increased photosynthesis efficiency, and further boosted antioxidant activities, and contents of *t*Zck and Sim, while minimizing Cd^2+^ levels in roots, leaves, and grains. The levels of O_2_^•−^, H_2_O_2_, MDA, and EL were also minimized, reflecting positively on growth and productivity. *t*Zck + Sim applied as SPr + LTr was highly effective in promoting antioxidants and photosynthesis machineries, minimizing oxidative stress biomarkers and Cd^2+^ levels, boosting tolerance to Cd stress, and improving wheat productivity under Cd stress.

## 1. Introduction

Security of food and nutrition is a global issue fundamental to the concept of sustainable human development [1]. Various contaminant types in agricultural soils cause deleterious effects on crop growth, yield, and quality [2,3,4]. Out of the pollutants present in the agricultural environment, heavy metals (HMs) can upset the natural balance of agroecology owing to their cumulative and eco-toxicity (having a non-degradable nature) to various living beings [2,5,6]. Different HMs, including cadmium (Cd), are non-essential micronutrients for plants and pose serious threats to their life [7,8]. Therefore, eco-remediation of HMs has become an inevitable prerequisite.

Cadmium (Cd) is an environmentally toxic metal that accumulates in agricultural soils worldwide. It mainly arises from anthropogenic sources, i.e., mining and smelting [9], irrigation with industrial effluents, adding phosphorus fertilizers and sewage sludge to soil, agricultural wastewater, and atmospheric sedimentation [10]. It can be transported directly from soil to plant edible parts, posing a hazard to human health through agro-food chains [11,12]. Cereal-based food products are the major Cd source, with more than 80% of the Cd in foods coming from cereals, followed by vegetables [13]. Long-term exposure to Cd toxicity may cause leaf chlorosis, root tip swelling, discoloration, torsion, necrosis, and lateral root growth retardation, reducing essential element uptake [14,15,16]. Even short-term exposure to Cd in soil stimulates oxidative stress through abnormal production of ROS (reactive oxygen species), leading to diminished seed germination and photosynthesis efficiency, damaged DNA and photosynthetic machinery, disrupted cyto-membranes, inhibited activity of antioxidant enzymes, and closed stomata, thus a reduction in growth and grain yield and quality [16,17,18,19].

Wheat is an essential strategic crop worldwide and the expansion of its cultivation contributes greatly to ensuring human food security. However, it is sensitive to various eco-stressors [20], including HMs stress [3,21,22,23]. It is a staple food crop and contributes to feeding more than 50% of the world’s population [24]. Additionally, in Cd-polluted soil, wheat plants can uptake a high Cd amount through the fibrous root system, translocate it to shoots, and finally accumulate it in their aerial parts, including grains [25]. Therefore, there is a pressing need to develop viable remedial strategies to minimize Cd absorption and accumulation in wheat grains.

The application of low-cost, eco-friendly practices to plants on HMs-contaminated soil to address the adverse influences of HMs, including Cd toxicity has received a lot of attention recently [4,16,25,26,27,28,29]. Noteworthy attempts have been made to minimize Cd level in food crops like wheat and minimize its availability in soil, including cultivation of low-Cd wheat genotypes [30], irrigation water management [31], application of nanomaterials, and amendments to plants and soils [25,32,33], application of microbial bioremediation [34], and use of natural biostimulants [4].

Plant growth regulators (PGRs) are synthesized naturally in plants to regulate plant growth under normal and stress conditions. PGRs increase plant branching and re-branching, as well as shoot and root growth. They also alter or stimulate maturing of fruits and the reproduction of plants. They play pivotal roles during stress conditions to mitigate abiotic stresses. They act as ROS-scavengers, thermoprotectants, photosynthesis- and metabolism-enhancers through the accumulation of stress proteins [35]. PGRs interconnect with complex signaling systems to balance responses to develop eco-friendly strains for satisfactory growth and productivity under stress conditions. They also support plants to develop complex mechanisms to detect external signals and can lead to an optimal response against stress conditions. Therefore, PGRs primarily control plant defense responses through both synergistic and antagonistic activities (termed crosstalk signals) [35].

Cytokinins (CKs), adenine-derived phytohormones, have divergent vital roles in regulating various physio-biochemical processes, i.e., cell differentiation and division, plant vasculature development, leaf senescence, as well as root and shoot meristematic activity, particularly under stress [36,37]. *Trans*-zeatin-type cytokinin (*t*Zck) is the major active form of CKs that are naturally biosynthesized in plant cells [38]. Based on classical bioassays of CKs, *t*Zck is existent and active in all higher plants, but *cis*-isomer is active only in fewer species of plants, although it is ubiquitous in all plant kingdoms [39]. The *t*Zck is known to be long-distance information carriers [40] and is mainly biosynthesized in the root vascular bundle and transported via the xylem shootward [41]. Positioning *t*Zck production is crucial for root and shoot meristematic activity, as it acts as a hormonal signaling transporter around its biosynthetic sites [40]. Based on previous reports, *t*Zck improves plant growth and regulates responses to stress [42,43,44], however, its endogenous production is often not sufficient to help plants effectively withstand abiotic stress. Consequently, the exogenous application of *t*Zck could be beneficial in upregulating plant tolerance to stress, including Cd [4,45].

On the other hand, silymarin (Sim) is the main bioactive compound, a polyphenolic flavonolignans complex mixture directly extracted from seeds, roots, stems, or leaves of *Silybum marianum* L. plants (*Asteraceae* family). It has been utilized to cure liver diseases [46]. More recently, it has been used alone or as an additive to enrich plant biostimulants to attenuate the adverse impacts of stress [4,47], being a secondary metabolite; a bioactive antioxidant. Its extract is comprised of six chemical bioactive flavonoids and flavonolignans; isosilychristins, isosilybins (A and B), silybinins or silibinins (A and B), silychristins, silydianins, and flavonoids (e.g., taxifolin) [48]. They make up 60 to 80% of the extract of *S. marianum* seeds and often account for 1 to 8% of the dry seed’s outer shell [49]. As a powerful antioxidant, exogenous supplementation of Sim can stimulate the performance of plants under stress by reinforcing their ROS-suppressing defense system thus providing considerable protection contra oxidative damage. As documented in a unique scientific report on maize [4], foliar Sim spraying at 0.5 mM was successful in repressing oxidative damage, upregulating plant defense mechanisms, antioxidant gene expressions, and reducing Cd toxicity stress.

As we know, no work has been realized on the influence of exogenous *t*Zck and Sim prepared as grain soaking + foliar spraying solutions and applied individually or in a combined mixture thereof on the plant’s physio-biochemistry, photosynthetic parameters, antioxidant defense capacity, and enzymatic activities of wheat growing on Cd-polluted soil. Based on previous research [4,42,43], we hypothesized that exogenous single or integrated treatments of *t*Zck + Sim would positively improve cellular antioxidant capacity that can protect the plant from the devastating influences of Cd. This research was planned to explore the influences of seed priming + leaf treatment with *t*Zck + Sim as plant activators on growth and yield traits, physio-biochemical and photosynthetic parameters, grain content of Cd, antioxidant defense capacity, and the activity of antioxidant enzymes of Cd-stressed wheat plants.

## 2. Materials and Methods

### 2.1. Plant Material, Experimental Description and Layout

Sterilized and certified wheat seeds (Sakha 93 cultivar) were purchased from the Agricultural Research Center, Egypt. The seeds were germinated in sterilized 12 cm-Petri dishes (10 seeds per dish) under suitable conditions (12 h/12 h light/darkness and 22 ± 2 °C) for 7 days. Appropriate plastic pots (40 × 38 cm in diameter × depth, respectively) filled with 12.5 kg of clean sand (free of ions) moistened with an appropriate nutrient solution [4,50] were utilized. The seedlings (germinated seeds) were transplanted, carefully, into the pots (10 each). Table S1 displays the composition of the nutrient solution, which was used for watering wheat seedlings/plants once every two days. Seeding/plant growth was controlled under the following conditions: 390 mE m^−2^ s^−1^ photon flux, 12/12 h dark/light, 16/20 °C and 68–72% relative humidity.

Starting at 2 weeks after transplanting (WaT), treatments with cadmium (Cd) (Sigma-Aldrich, St. Louis, MO, USA) were applied in the nutrient solution to the seedlings/plants. Cd at 0.6 mM (using CdSO_4_) was chosen based on a preliminary study, as 15 irrigations with 0.6 mM was more harmful to wheat plants than 0.2 or 0.4 mM. Plants were killed at a concentration of 0.8 mM when used in 15 irrigations (data not shown). An 8-h grain soaking and two foliar sprays were applied using trans-zeatin-type cytokinin (*t*Zck) at 0.05 mM and silymarin (Sim; Sigma-Aldrich, St Louis, MO, USA) at 0.5 mM. The *t*Zck and Sim levels were applied singly or in a combined solution. The spraying solutions were applied 7 days after the first irrigation with the nutrient solution polluted with 0.6 mM Cd. One more foliar spray was performed 14 days later. Another preliminary experiment was conducted to set the best levels; 0.05 and 0.5 mM of tZck and Sim, respectively, for use in the main study (data not shown). A few drops of Tween-20 were added to the spraying solutions as a surfactant. To keep Cd level at 0.6 mM in the growing medium, the Perkin-Elmer Inductively Coupled Plasma (Optima 3300DV ICP-MS, Mundelein, IL, USA) was used continuously. Irrigation with the nutrient solution containing 0.6 mM was applied 15 times starting 14 days after transplantation. Table 1 presents the 8 study treatments (20 pots = 4 replicates × 5), which were arranged in a completely randomized design (CRD).

Plant samples (4 random pots from each treatment; 1 pot from each replicate) were taken 50 days after transplantation to determine growth traits, photosynthetic efficiency indices, levels of markers of oxidative stress and their consequences. In addition to the determination of Cd levels in different plant parts, the activities of the antioxidant system components and antioxidant redox states, as well as the contents of *t*Zck and Sim were determined. Then, the experiments continued until harvest (130 days after transplantation) to determine yield traits.

### 2.2. Traits of Wheat Growth and Yield

The shoot length of each plant was recorded utilizing a 1 m graduated ruler. Plant leaves were counted and a leaf Area Meter (LI-3100C, Lincoln, NE, USA) was utilized to scan for plant leaf area. Shoot dry weight was recorded after drying at 70 °C for 2 days. At harvest, spikes were counted to record the number of spikes per plant. Then the spikes were extracted to compute the number of grains per spike, as well as the weight of grains per pot (per 10 plants) and the weight of 1000 seeds.

### 2.3. Determinations of Physiological-Biochemical Indices

For all of these determinations, uniform leaves (the top two full-grown leaves) on each plant without midribs (blades only) were utilized. The Konrad et al. [50], Wellburn [51], and Avron [52] procedures were practiced to evaluate the instantaneous efficiency of carboxylation (iEC; µmol m^−2^ s^−1^), contents of leaf chlorophylls and carotenoids (mg g^−^^1^ FW), and photochemical activity (KCN technique), respectively. The contents of Cd^+2^ (mg kg^−1^ DW) in the roots, leaves, and yielded grains were evaluated depending on the Chapman and Pratt [53] procedures. The content of silymarin (Sim) was evaluated by applying the Arampatzis et al. [54,55] methods using HPLC system. Cis- and trans-zeatin-type cytokinin and total cytokinins were extracted and analyzed applying the Novák et al. [56] procedures.

### 2.4. Oxidative Stress Biomarker Levels and Their Consequences and Antioxidant System Components

For all of these determinations, uniform leaves (the top two full-grown leaves) on each plant without midribs (blades only) were utilized. The contents of O_2_^•−^ (A_580_ g^−1^ FW), H_2_O_2_ and MDA (µmol g^−1^ FW) were evaluated by applying the procedures of Kubis [57], Velikova et al. [58], and Heath and Packer [59], respectively. The procedures of Rady [60] were followed to evaluate the leakage of ions from leaf tissue. The contents of proline (µmol g^−1^ DW), AsA (µmol g^−1^ FW) and its redox capacity, and GSH (µmol g^−1^ FW) and its redox capacity were evaluated by applying the full procedures of Bates et al. [61], Kampfenkel and Van Montagu [62], and Griffth [63], respectively.

A fresh leaf blade (0.5 g) and K-P buffer (pH = 7.8) were utilized for obtaining the enzymatic extract. In the extract, the procedures of Bradford [64] were applied to evaluate total soluble protein content. The activities of SOD, CAT, APX and GR were assayed following the detailed procedures of Kono [65], Aebi [66], and Rao et al. [67].

### 2.5. Data Analysis

Applying the statistical software Statistix^®^, version 8.1 (Copyright 2005, Analytical Software, NorthEdge, Seattle, WA, USA), the analysis of the resulting data was performed with a two-way ANOVA design [with two levels of cadmium stress (−Cd and +Cd) × four stimulus treatments (control, *t*Zck, Sim, and *t*Zck + Sim) eight combination treatments] with four randomized blocks each [68]. Means computed for treatments were compared by applying the LSD Test at *p* ≤ 0.05.

## 3. Results

### 3.1. Wheat Growth and Yield, and Leaf Photosynthetic Efficiency in Response to tZck and/or Sim

**Figure 1:**
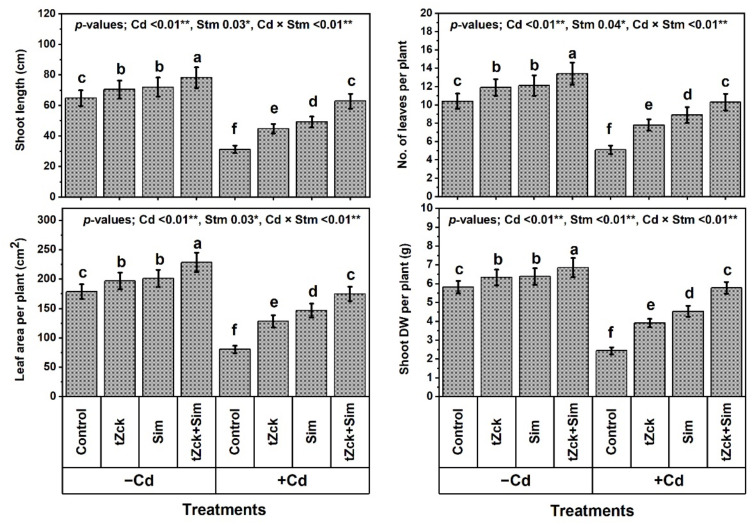
Growth traits of wheat (cv. Sakha 93) treated with two stimulators (Stm) i.e., silymarin (Sim; 0.5 mM) and *trans*-zeatin-type cytokinin (*t*Zck; 0.05 mM) under cadmium (Cd; 0.6 mM) stress. The same letters with mean values ± SE in each plot indicate non-significant differences based on the LSD test (*p* ≤ 0.05). * and ** refer to significant difference at *p* ≤ 0.05 and *p* ≤ 0.01, respectively. *t*Zck; *trans*-zeatin-type cytokinin, Sim; silymarin, −Cd; without cadmium treatment, +Cd; cadmium treatment and Stm; stimulator.

**Figure 2:**
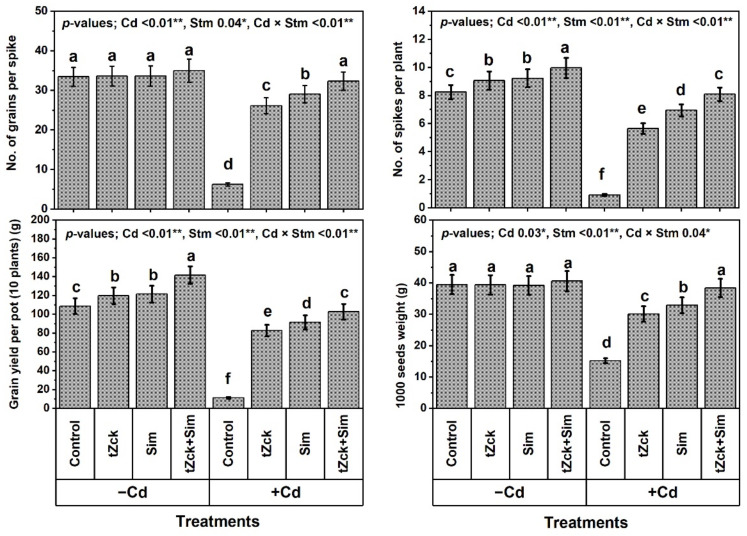
Yield traits of wheat (cv. Sakha 93) treated with two stimulators (Stm) i.e., silymarin (Sim; 0.5 mM) and *trans*-zeatin-type cytokinin (*t*Zck; 0.05 mM) under cadmium (Cd; 0.6 mM) stress. The same letters with mean values ± SE in each plot indicate non-significant differences based on the LSD test (*p* ≤ 0.05). * and ** refer to significant difference at *p* ≤ 0.05 and *p* ≤ 0.01, respectively. *t*Zck; *trans*-zeatin-type cytokinin, Sim; silymarin, −Cd; without cadmium treatment, +Cd; cadmium treatment and Stm; stimulator.

**Figure 3:**
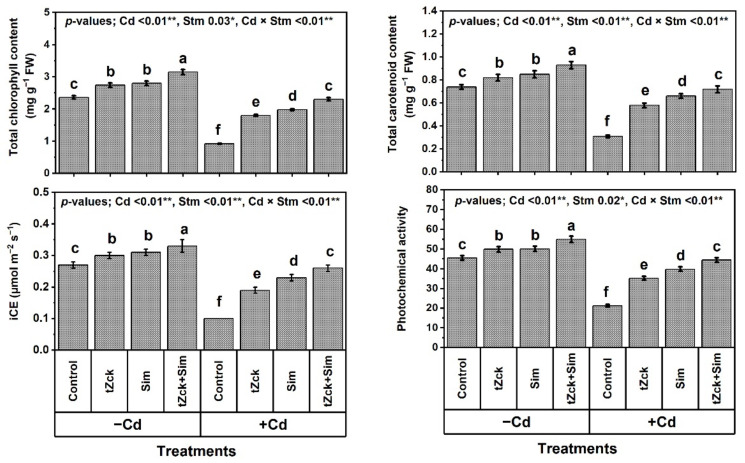
Leaf photosynthetic efficiency (total chlorophyll and carotenoid contents, instantaneous carboxylation efficiency; iCE, and photochemical activity) of wheat (cv. Sakha 93) treated with two stimulators (Stm) i.e., silymarin (Sim; 0.5 mM) and *trans*-zeatin-type cytokinin (*t*Zck; 0.05 mM) under cadmium (Cd; 0.6 mM) stress. The same letters with mean values ± SE in each plot indicate non-significant differences based on the LSD test (*p* ≤ 0.05). * and ** refer to significant difference at *p* ≤ 0.05 and *p* ≤ 0.01, respectively. *t*Zck; *trans*-zeatin-type cytokinin, Sim; silymarin, −Cd; without cadmium treatment, +Cd; cadmium treatment and Stm; stimulator.

Under the non-Cd-stressed conditions, grain soaking + foliar spraying two times using 0.05 mM *t*Zck or 0.5 mM Sim significantly increased growth traits (shoot length by 8.6 or 11.1%, plant leaf no. by 14.4 or 16.3%, plant leaf area by 10.2 or 12.5% and shoot DW by 8.9 or 9.6%, respectively), yield traits (no. of spikes per plant by 10.0 or 11.9% and grain yield by 10.2 or 11.7%, respectively), photosynthetic pigments (total chlorophyll by 16.1 or 18.6% and total carotenoid by 10.8 or 14.9%, respectively), and leaf photosynthetic efficiency (instantaneous carboxylation efficiency; iCE by 11.1 or 14.8% and photochemical activity by 9.7 or 10.1%, respectively) of wheat plants compared to control (Figure 1, Figure 2 and Figure 3). 

Grain soaking + foliar spraying two times using 0.05 mM *t*Zck + 0.5 mM Sim was a more effective treatment, increasing shoot length by 20.7%, plant leaf no. by 28.8%, plant leaf area by 27.8%, shoot DW by 17.9%, plant spike no. by 20.9%, grain yield by 30.3%, total chlorophyll by 33.5%, total carotenoid by 25.7%, iCE by 22.2%, and photochemical activity by 20.7% compared to the control. Noticeably, wheat plants under Cd treatment exhibited a more deteriorated effect compared to control, decreasing shoot length, plant leaf no., plant leaf area, shoot DW, no. of grains per spike, plant spike no., grain yield, 1000 seeds weight, total chlorophyll, total carotenoid, iCE, and photochemical activity by 51.9, 51.0, 55.0, 58.1, 81.4, 88.8, 89.7, 61.5, 61.0, 58.1, 63.0 and 53.4%, respectively. Compared to Cd (0.6 mM) treatment, grain soaking + foliar spraying two times with 0.05 mM *t*Zck or 0.5 mM Sim significantly increased all the former traits, whereas 0.05 mM *t*Zck + 0.5 mM Sim treatment was more efficacious, increasing all the same traits significantly by 101.3, 102.0, 117.4, 136.9, 421.0, 778.3, 817.9, 152.6, 150.0, 132.3, 160.0 and 109.4%, respectively. Interestingly, there was no significant (statistically) difference between wheat plants applied with Cd + (0.05 mM *t*Zck + 0.5 mM Sim) and control regarding growth, productivity, photosynthetic pigments, and efficiency.

### 3.2. Markers of Oxidative Stress Levels and Their Consequences in Response to tZck and/or Sim

**Figure 4:**
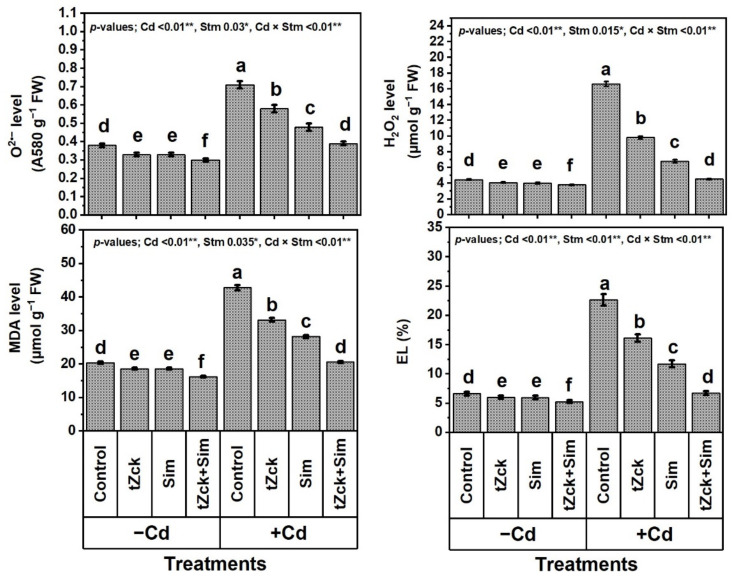
Markers of oxidative stress [superoxide (O_2_^•^^−^) and hydrogen peroxide (H_2_O_2_)] and their consequences [lipid peroxidation as malondialdehyde (MDA) and electrolyte leakage (EL)], cadmium (Cd) level, free proline content, ascorbate (AsA) and glutathione (GSH) levels and their redox capacity (%) of wheat (cv. Sakha 93) treated with two stimulators (Stm) i.e., silymarin (Sim; 0.5 mM) and *trans*-zeatin-type cytokinin (tZck; 0.05 mM) under Cd (0.6 mM) stress. The same letters with mean values ± SE in each plot indicate non-significant differences based on the LSD test (*p* ≤ 0.05). * and ** refer to significant difference at *p* ≤ 0.05 and *p* ≤ 0.01, respectively. *t*Zck; *trans*-zeatin-type cytokinin, Sim; silymarin, −Cd; without cadmium treatment, +Cd; cadmium treatment and Stm; stimulator.

Under Cd stress-free conditions, markers of oxidative stress (O_2_^•−^ and H_2_O_2_) and their consequences (i.e., lipid peroxidation as MDA and EL) were significantly decreased due to grain soaking + foliar spraying two times with 0.05 mM *t*Zck, 0.5 mM Sim, or even with 0.05 mM *t*Zck + 0.5 mM Sim, which was the best treatment, compared to control (Figure 4). 

With the addition of 0.6 mM Cd to the nutrient irrigation solution, the levels of O_2_^•−^, H_2_O_2_, MDA, and EL increased significantly by 86.8%, 274.3%, 109.8%, and 242.0%, respectively. Compared with Cd (0.6 mM) treatment, all of the above traits were remarkably decreased due to grain soaking + foliar spraying two times with *t*Zck (0.05 mM) or Sim (0.5 mM); however, *t*Zck (0.05 mM) + Sim (0.5 mM) treatment was more efficacious, with all the former traits decreased by 45.1%, 72.8%, 51.9%, and 70.4%, respectively. Wheat plants exposed to Cd treatment were able to minimize the markers of oxidative stress, which was mirrored in the notable declines of oxidative stress biomarkers and their consequences (especially H_2_O_2_ and EL) by the co-treatment with 0.05 mM *t*Zck + 0.5 mM Sim as grain soaking and foliar spraying two times.

### 3.3. Root, Leaf and Yielded Grain Cd^2+^ Contents in Response to tZck and/or Sim

**Figure 5:**
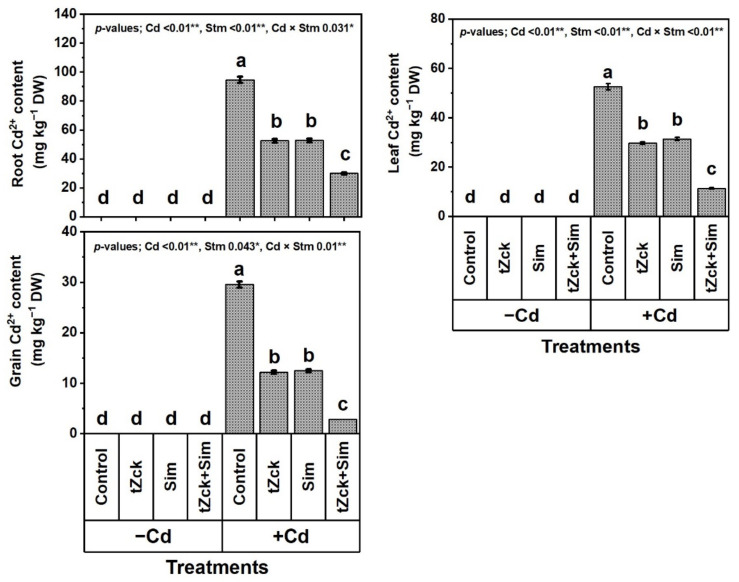
Root, leaf and grain cadmium (Cd) contents of wheat plants (cv. Sakha 93) treated with two stimulators (Stm) i.e., silymarin (Sim; 0.5 mM) and *trans*-zeatin-type cytokinin (tZck; 0.05 mM) under Cd (0.6 mM) stress. The same letters with mean values ± SE in each plot indicate non-significant differences based on the LSD test (*p* ≤ 0.05). * and ** refer to significant difference at *p* ≤ 0.05 and *p* ≤ 0.01, respectively. *t*Zck; *trans*-zeatin-type cytokinin, Sim; silymarin, −Cd; without cadmium treatment, +Cd; cadmium treatment and Stm; stimulator.

Under the non-stressed conditions, no Cd^2+^ was detected in wheat roots, leaves, and yielded grains in all treatments including control (Figure 5).

Remarkably, wheat plants under Cd treatment exhibited a severe deterioration effect compared to control, and the root, leaf, and grain Cd^2+^ contents were detected at 94.8, 2. and 29.6 mg kg^−1^ DW, respectively. Compared to Cd (0.6 mM) treatment, grain soaking + foliar spraying two times with 0.05 mM *t*Zck or 0.5 mM Sim significantly decreased the root, leaf and grain Cd^2+^ contents by 44.4 or 44.2%, 43.3 or 40.3% and 58.8 or 57.8%, respectively, whereas 0.05 mM *t*Zck + 0.5 mM Sim treatment was more efficacious, decreasing the root, leaf and grain Cd^2+^ contents by 68.2, 78.3 and 90.2%, respectively. Interestingly, the decrease in Cd^2+^ content was higher in grains than in leaves, and the decrease was higher in leaves than in roots.

### 3.4. Free Proline Content (FPC), Capacity of Ascorbate (AsA) and Glutathione (GSH), Silymarin (Sim) and Trans-Zeatin-Type Cytokinin (tZck) Contents in Response to tZck and/or Sim

**Figure 6:**
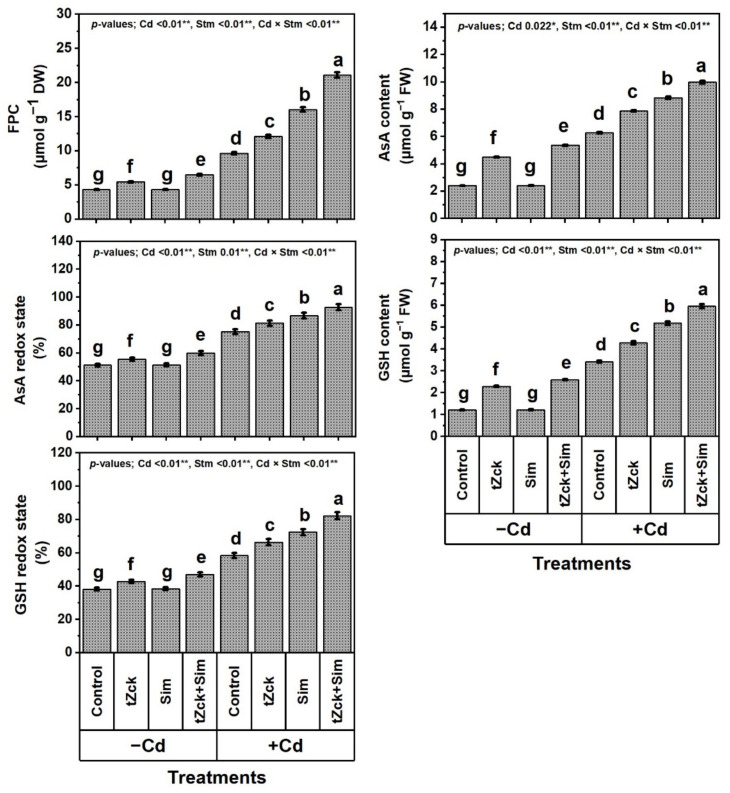
Free proline content (FPC), levels of ascorbate (AsA) and glutathione (GSH) and their redox states (%) of wheat plants (cv. Sakha 93) treated with two stimulators (Stm) i.e., silymarin (Sim; 0.5 mM) and *trans*-zeatin-type cytokinin (tZck; 0.05 mM) under Cd (0.6 mM) stress. The same letters with mean values ± SE in each plot indicate non-significant differences based on the LSD test (*p* ≤ 0.05). * and ** refer to significant difference at *p* ≤ 0.05 and *p* ≤ 0.01, respectively. *t*Zck; *trans*-zeatin-type cytokinin, Sim; silymarin, −Cd; without cadmium treatment, +Cd; cadmium treatment and Stm; stimulator.

**Figure 7:**
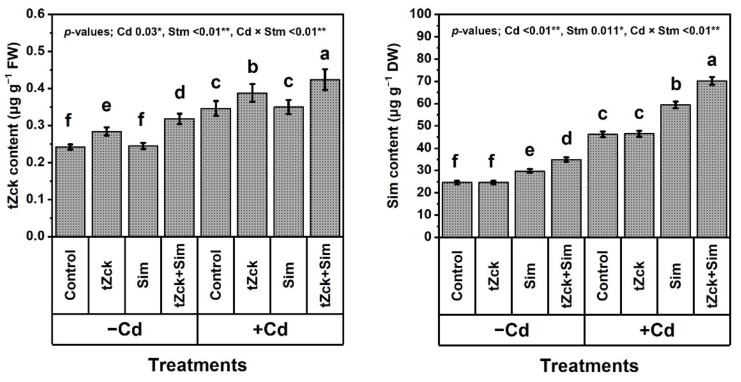
Silymarin (Sim) and *trans*-zeatin-type cytokinin (*t*Zck) contents of wheat (cv. Sakha 93) treated with two stimulators (Stm) i.e., Sim (0.5 mM) and tZck (0.05 mM) under cadmium (Cd; 0.6 mM) stress. The same letters with mean values ± SE in each plot indicate non-significant differences based on LSD test (*p* ≤ 0.05). * and ** refer to significant difference at *p* ≤ 0.05 and *p* ≤ 0.01, respectively. *t*Zck; *trans*-zeatin-type cytokinin, Sim; silymarin, −Cd; without cadmium treatment, +Cd; cadmium treatment and Stm; stimulator.

Compared with control, the *t*Zck and/or Sim treatment positively and significantly affected the FPC, contents, and redox capacity of AsA and GSH, as well as Sim and *t*Zck contents in wheat plants (Figure 6 and Figure 7).

Grain soaking + foliar spraying two times with 0.05 mM *t*Zck + 0.5 mM Sim was more effective, it remarkably elevated FPC by 50.0%, AsA level by 122.4%, AsA redox capacity by 17.0%, GSH level by 114.0%, GSH redox capacity by 23.0%, *t*Zck content by 31.4%, Sim content by 41.9%. The increase in *t*Zck and Sim contents by 17.4% and 21.1% were considerable under grain soaking + foliar spraying two times with *t*Zck (0.05 mM) and Sim (0.5 mM) treatments, respectively, compared with control. Under no use of *t*Zck and/or Sim, Cd (0.6 mM) treatment resulted in an increase in levels of FPC by 123.1%, AsA by 160.6%, GSH by 182.6%, *t*Zck by 43.0%, and Sim by 87.8%, in addition to cellular redox states of AsA by 46.9% and GSH by 52.6% compared to control. Compared to Cd treatment, plants applied with *t*Zck or Sim showed a noticeable increase in all the above traits. However, the highest (significant) values for the above traits were noticed under *t*Zck + Sim treatment. This combined treatment (Cd + *t*Zck + Sim) mitigated the negative impacts of Cd stress by showing 119.1, 58.9, 23.4, 74.3, 41.0, 22.5, 51.9% boost in FPC, levels, and cellular redox states of AsA, GSH, as well as *t*Zck and Sim, compared with Cd treatment. Under co-application of Cd + (tZck + Sim), 0.05 mM *t*Zck + 0.5 mM Sm qualified wheat plants to be free from Cd toxic influences by helping plants to accumulate more cellular antioxidants, i.e., FPC, AsA, GSH, *t*Zck, and Sim to efficiently defend against oxidative stress biomarkers.

### 3.5. Enzymatic Activities in Response to tZck and/or Sim

**Figure 8:**
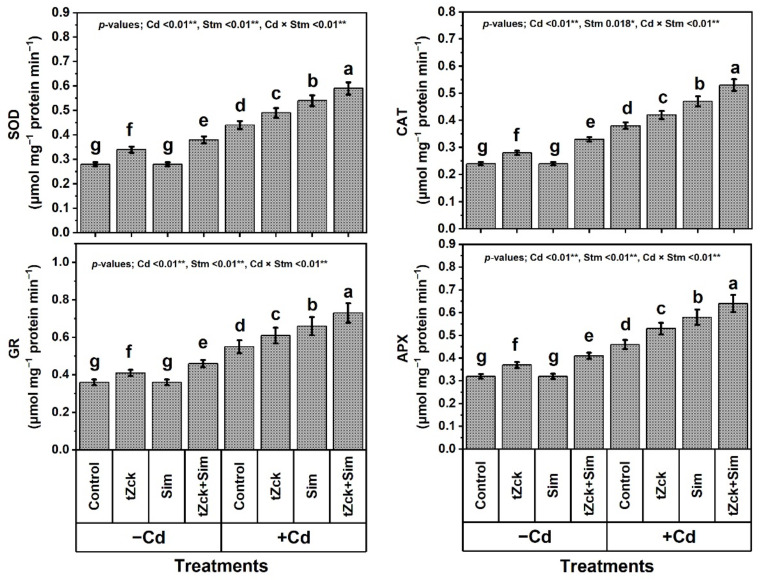
Enzyme activities of wheat (cv. Sakha 93) treated with two stimulators (Stm) i.e., silymarin (Sim; 0.5 mM) and *trans*-zeatin-type cytokinin (*t*Zck; 0.05 mM) under cadmium (Cd; 0.6 mM) stress. The same letters with mean values ± SE in each plot indicate non-significant differences based on the LSD test (*p* ≤ 0.05). SOD, superoxide dismutase; CAT, catalase; APX, ascorbate peroxidase; and GR, glutathione reductase. * and ** refer to significant difference at *p* ≤ 0.05 and *p* ≤ 0.01, respectively. *t*Zck; *trans*-zeatin-type cytokinin, Sim; silymarin, −Cd; without cadmium treatment, +Cd; cadmium treatment and Stm; stimulator.

Findings of this study revealed that the activities of SOD, CAT, APX, and GR (Figure 8) were significantly increased due to *t*Zck and/or Sim treatment under stress-free conditions. The best enzymatic activities were obtained by soaking grains + foliar spraying with 0.05 mM *t*Zck + 0.5 mM Sim. This best *t*Zck + Sim treatment increased the activity of SOD by 35.7%, CAT by 37.5%, APX by 28.1%, and GR by 27.8% compared with control. Cd (0.6 mM) treatment without any stimulating applications showed a significant increment in the activity of SOD by 57.1%, CAT by 58.3%, APX by 43.8%, and GR by 52.8%, respectively, compared with healthy (stress-free) plants. 

Compared with Cd (0.6 mM) treatment, plants applied with *t*Zck (0.05 mM) and/or Sim (0.5 mM) treatment exhibited significant improvements in enzyme activities. However, 0.05 mM *t*Zck + 0.5 mM Sim displayed the most effective treatment, boosting SOD, CAT, APX and GR activities by 34.1%, 39.5%, 39.1%, and 32.7%, respectively. Upon exogenous supplementation of 0.05 mM *t*Zck + 0.5 mM Sim as grain soaking + foliar spraying, the stressed wheat plants can maximize the activities of defense-related enzymes (SOD, CAT, APX, and GR) to overcome biomarkers of oxidative stress, recovering Cd-related toxicity and damage to different cellular organelles.

### 3.6. Relationship between Treatments and the Parameters Studied and Correlation Analysis

**Figure 9:**
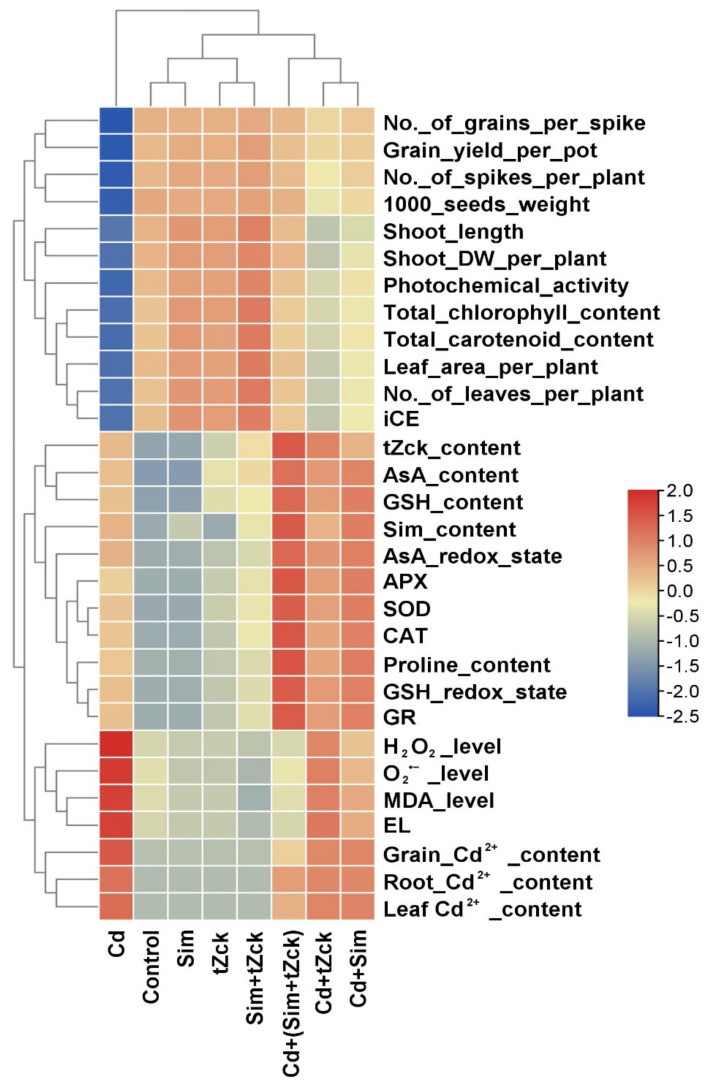
Heatmap graph shows the analysis of hierarchical clustering among the different studied treatments and parameters. The colors represent variations in the obtained data. iCE; instantaneous carboxylation efficiency, O_2_^•^^−^; superoxide, H_2_O_2;_ hydrogen peroxide level, MDA; malondialdehyde level, EL; electrolyte leakage, Cd; cadmium content, AsA; ascorbate level, GSH; glutathione, Sim; Silymarin, *t*Zck; *trans*-zeatin-type cytokinin contents, SOD; superoxide dismutase, CAT; catalase, APX; ascorbate peroxidase and GR; glutathione reductase.

The hierarchical clustering and Pearson’s correlation analyses were performed to explore the relationship among observed parameters in wheat (cv. Sakha 93) plants applied with *t*Zck (0.05 mM) and Sim (0.5 mM) exposed to 0.6 mM Cd stress. The hierarchical clustering analysis divided the experimental treatments into three groups in which the Cd^+^ stress treatment without *t*Zck or Sim applications was divided in the first group. The second group included the *t*Zck, Sim, *t*Zck + Sim, and control, while Cd + tZck, Cd + Sim, and Cd + (tZck + Sim) were clustered together in the third group that had better performance compared to Cd^+^ stress treatment without *t*Zck or Sim applications (Figure 9).

**Figure 10:**
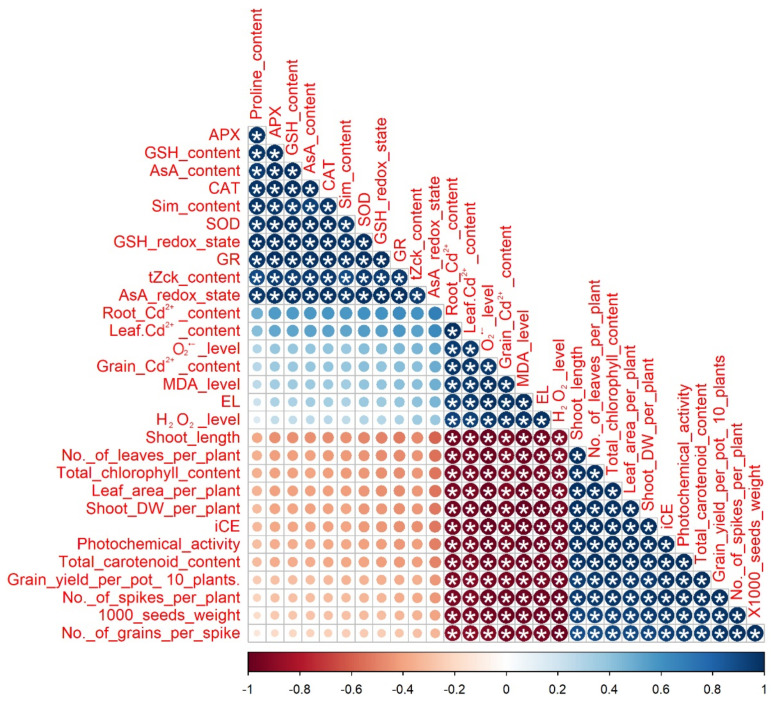
Pearson’s correlation analysis among the different studied parameters. The colors represent variations in the obtained data. iCE; instantaneous carboxylation efficiency, O_2_^•^^−^; superoxide, H_2_O_2_; hydrogen peroxide level, MDA; malondialdehyde level, EL; electrolyte leakage, Cd; cadmium content, AsA; ascorbate level, GSH; glutathione, Sim; silymarin, *t*Zck; *trans*-zeatin-type cytokinin contents, SOD; superoxide dismutase, CAT; catalase, APX; ascorbate peroxidase and GR; glutathione reductase. * *p* ≤ 0.05.

Pearson’s correlation analysis declared a significant positive correlation (*p* ≤ 0.05) between total carotenoid content, photochemical activity, iCE, shoot dry weight, leaf area, total chlorophyll content, plant leaf no. and shoot length with the grain yield, plant grain no., plant spike no. and 1000-seed weight. O_2_^•−^, H_2_O_2_, MDA, EL levels, leaf Cd^2+^, root Cd^2+,^ and grain Cd^2+^ contents had a negative (significant) correlation (*p* ≤ 0.05) with the above-mentioned traits (Figure 10). Moreover, a positive correlation (*p* ≤ 0.05) was found between states of ASA redox, GSH redox, and APX, CAT, SOD, and GR activities with *t*Zck, Sim, ASA, and GSH contents.

## 4. Discussion

In recent decades, unwise human activities have exacerbated the release of metallic pollutants, including Cd, into agricultural soils in many regions of the world [69,70]. Cd-contaminated soil represents environmental stress that hinders crop plant growth and productivity [3,4]. The accumulation of Cd in edible plant parts in Cd-contaminated soil brings grave danger to human health when it enters food chains [2] through the soil-plant-food route. In the present study, Cd set at 0.6 mM in the nutritive watering solution drastically reduced wheat morpho-physiological and yield traits (Figure 1, Figure 2 and Figure 3), while it abundantly elevated oxidative stress biomarkers (O_2_^•−^, H_2_O_2_, MDA, and EL) and Cd^2+^ contents in roots, leaves and yielded grains (Figure 4 and Figure 5). Osmoprotectant levels (FPC, AsA, and GSH) and antioxidant redox capacities (Figure 6), as well as leaf *t*Zck and Sim contents (Figure 7) and enzyme activities (Figure 8), were also elevated compared to non-stressed plants. However, soaking grains + foliar spraying two times with 0.05 mM *t*Zck and/or 0.5 mM Sm stimulated minimization of Cd toxicity in plants, improving growth and yield, leaf photosynthetic pigments, and leaf photosynthetic efficiency, reducing oxidative stress biomarkers and Cd contents (in roots, leaves and yielded grains), and positively modifying all antioxidative (i.e., enzymatic and non-enzymatic) defense machineries. These results concur with those of Alharby et al. [4], Alharby et al. [42], Azzam et al. [43], Salman et al. [47], and Desoky et al. [71], who reported that treating plants with Sim or *t*Zck as grain soaking or foliar application regulated the plant’s adaptive responses via physio-biochemical, metabolic and molecular modulation under different abiotic and biotic stresses, including Cd stress.

The findings of this investigation revealed that Cd stress induced an oxidative burst, which then led to the accumulation of ROS in cellular structures of wheat plant tissues, resulting in growth retardation [72,73]. Moreover, Cd toxicity stress reduced biomass production due to the severe impairment of root growth, as roots accumulated Cd in higher concentrations compared to shoots (Figure 5). Like our findings (Figure 5), Zeshan et al. [16] reported that plant roots grown in a Cd-polluted soil uptake and amass a higher Cd level than their shoots. A decline in biomass accumulation corresponds to cellular oxidative damage, limited nutrient supply, and Cd-associated phytotoxicities. Otherwise, to reform and minimize damage caused by exposure to abiotic stresses such as Cd toxicity stress, plants develop their antioxidant complex systems [74]. However, endogenously antioxidant components are frequently insufficient to counteract the damaging impacts of environmental stressors [42,71]. Thus, it is very necessary to support plants under long-term abiotic stresses, including Cd, by treating them with exogenous substances such as *t*Zck and Sim for their survival and to maintain a high production level.

After the grains are soaked, the *t*Zck (as an adenine-derived phytohormone) and/or Sim (as a secondary metabolite) may easily pass into wheat grains, allowing them to germinate quickly and vigorously, leading to healthy seedlings that can withstand Cd-contaminated soil stress conditions [4]. Furthermore, foliar spraying of 0.05 mM *t*Zck and/or 0.5 mM Sim co-applied with grain soaking, under control (no stress) or Cd stress conditions, collaboratively increased photosynthetic pigments (chlorophylls and carotenoids), improved photosynthesis capacity (iCE and photochemical activity) and other physio-biochemical traits, all of which positively affected plant growth, particularly dry matter production, and grain yield with minimal Cd content (Figure 1, Figure 2, Figure 3, Figure 4, Figure 5, Figure 6, Figure 7, Figure 8, Figure 9 and Figure 10). These improvements in growth may be due to the physiological role of *t*Zck and/or Sim in modulating the defensive responses to Cd stress through many protective mechanisms, including the regulation of defense-related genes and other phytohormones like salicylic acid (Jiang et al. [75] on rice), which have been reported to be CKs-responsive hormones [76]. Better shoot length and no. of leaves per plant resulting from *t*Zck-applied singly or together with Sim implicated in an increment in plant leaf area, attended by increased photosynthetic pigments, they all boosted photosynthetic efficiency (iCE and photochemical activity). Flavonoids and polyphenolic compounds (contained in Sim; [48]) supply stress protection owing to their actions as ROS scavengers and resistance to HMs toxicity stress. Flavonoids, chemically bioactive compounds, also inhibit cell-to-cell polar auxin transport and induce localized auxin pileup in the plant system [77]. These positive outcomes were favorably reflected in dry biomass accretion (Figure 1, Figure 2 and Figure 3). All these favorable outcomes were obtained by *t*Zck and/or Sim due to the suppressed levels of the oxidative stress markers (O_2_^•−^, H_2_O_2_, MDA, and EL) and Cd^2+^ contents in different plant parts, including grains (Figure 4 and Figure 5). 

The levels of ROS produced within stressed plant cells are controlled by both enzymatic and non-enzymatic antioxidant defense systems. Synthesis and pileup of different osmoprotective compounds and cytosolic compatible solutes are integral defensive mechanisms against oxidative stress stimulated by Cd toxicity stress [71]. Accumulation of proline as an osmoprotectant with AsA and GSH as low-molecular-weight metabolites under Cd stress conditions protects stressed plants by adjusting the cytosol and vacuole osmotic strengths inside the cells, as well as osmotic pressure of the outer environment [78,79,80,81]. In the current study, *t*Zck and/or Sim application improved the accumulation of FPC, as well as AsA and GSH and their redox capacities of control (stress-free) or Cd stress treatment. In this respect, Alharby et al. [4] and Azzam et al. [43] stated an increase in antioxidants (proline, AsA, and GSH) with suppressed ROS levels in maize plants exogenously treated with *t*Zck or Sim under Cd toxicity stress. These positive results could be attributed to *t*Zck-mediated increment in proline metabolism through catabolism and anabolism routes of ProDH and P5CS, respectively, which stimulate the higher and lower activity of ProDH and P5CS, respectively, to balance proline levels in plant cells [82]. Proline scavenges ROS and maintains the thylakoid biomembrane integrity [83]. AsA may be involved in cellular vacuolization, cellular wall extension, and then root system elongation, and plant cell cycle regulation under stress conditions as well [84]. Al-Hakimi and Hamada [85] showed that increasing AsA boosted the levels of pectin and cellulose in the cell wall of wheat plants. These cell wall components contain various functional groups; mercapto (−SH), hydroxyl (−OH), and carboxyl (−COOH), which can bind to Cd^2+^, hindering its uptake and mobility and promoting its compartmentalization under Cd stress [86]. GSH is also an essential non-enzymatic antioxidant and a circular redox buffer that affects defense-related gene expressions [87], and cellular redox homeostasis during cell division and elongation [88]. With *t*Zck and/or Sim treatment, considerable enhancements of these osmoprotectants and apoplastic antioxidants can provide efficient stress protection mechanisms to boost plant tolerance contra Cd-induced oxidative stress.

Antioxidant enzymes, including SOD, CAT, APX, and GR [89], provide another line of defense in plants against abiotic stresses, including Cd-contaminated soil. Our results exhibited that SOD, CAT, APX, and GR activities were increased with Cd-stimulated oxidative stress and then further increased under the application of *t*Zck and/or Sim (Figure 8). A similar trend was obtained by Guo et al. [90], Alharby et al. [4], Alharby et al. [42], and Desoky et al. [71]. Antioxidants enable plants to scavenge over-generated ROS catalyzed by Cd stress by minimizing the levels of O_2_^•−^, H_2_O_2_, MDA, and EL (Figure 4), as well as the contents of Cd^2+^ in different plant parts (Figure 5). SOD catalytically converts O_2_^•−^ radicals, which are produced in plant tissues under stress, into O_2_ and H_2_O_2_, a potent oxidant that is avoided by the AsA–GSH pathway. Hydroxyl radicals (OH^•^) are other hazardous reactive and harmful oxides and can react, indiscriminately, with all large cellular biomolecules. Both SOD and CAT enzymes can inhibit or minimize the production of OH^•^ by integrating their roles [91,92]. H_2_O_2_ levels are brought down by APX, and GSH and AsA contents are maintained by GR, leading to cellular redox homeostasis [93]. Given the variety of the bioactive components (i.e., isosilychristins, isosilybins, silybinins, silychristins, silydianins, and flavonoids) included in Sim as a central stimulator, it is a sustainable defensive strategy to treat wheat plants as grain soaking or foliar spraying for healthy growth, conferring wheat plants more protection against Cd stress [4,45]. The potential positive roles of Sim exogenously applied, as grain soaking + foliar spraying in improving wheat plant performance in response to Cd stress has not been fully reported yet. Sim has only been shown to improve plant productivity since it accumulates (Figure 7) under environmental stress conditions [94] to boost plant self-defense systems in accordance with our results. In this context, Afshar et al. [95] stated that Sim is considered a potent antioxidant, thus its role in enhancing plant tolerance to stress is attributable to its higher capacity as an antioxidant.

All the antioxidant mechanisms described above helped minimize Cd^2+^ contents in Cd-stressed wheat roots and leaves, and thus in grains harvested as a result of foliar spraying of 0.05 mM *t*Zck + 0.5 mM Sim co-applied with soaking seeds in these stimulators (*t*Zck + Sim) (Figure 5). This finding may be due to excessive compartmentalization of Cd^2+^, which allows for less Cd^2+^ influx from roots to leaves and further less influx from leaves to grains. As a consequence, the grains collected 2.9 mg per kg (2.9 ppm), which is close to the permissible limit for cereal grains [23,96].

## 5. Conclusions

In this study, the impacts of Cd-contaminated soil were assessed on wheat growth, yield, and physio-biochemical indices. Our findings depicted that Cd stress considerably decreased wheat growth and productivity traits, and photosynthesis pigments and efficiency. Oxidative stress biomarkers, enzymatic and non-enzymatic antioxidants, redox states, and contents of *t*Zck, Sim, and Cd^2+^ in roots, leaves, and harvested grains were increased compared to non-stressed plants. However, grain soaking + foliar spraying two times with 0.05 mM *t*Zck + 0.5 mM Sim provided an effective viable strategy (bypassing *t*Zck or Sim alone) to mitigate the destructive influences of Cd stress. With superiority when integrating both *t*Zck and Sim, treated wheat plants exhibited better tolerance to the examined Cd stress and increased photosynthesis pigments and efficiency by minimizing ROS levels via enhanced various antioxidants (proline, AsA, GSH, *t*Zck, Sim, SOD, CAT, APX, and GR), and redox capacities, as well as via decreased levels of O_2_^•−^, H_2_O_2_, MDA, EL, and Cd^2+^ (in different plant parts, especially grains). Finally, our findings suggest that grain soaking + foliar spraying two times with 0.05 mM *t*Zck + 0.5 mM Sim can be efficiently used to enhance Cd stress tolerance and improve the growth and productivity of wheat under Cd-polluted water or soil conditions. More detailed studies are required, in this regard, to further understand the underlying defense mechanisms mediated by *t*Zck and Sim, which confer greater tolerance to the stress-exposed plant.

## Figures and Tables

**Table 1:** Descriptions of study treatments.

Cd	Stm	Description
−Cd	Control	No stress; grain soaking + 2 foliar sprays using distilled water.
	*t*Zck	Grain soaking + 2 foliar sprays using 0.05 mM *t*Zck.
	Sim	Grain soaking + 2 foliar sprays using 0.5 mM Sim.
	*t*Zck + Sim	Grain soaking + 2 foliar sprays using 0.05 mM *t*Zck + 0.5 mM Sim.
+Cd	Control	Irrigating wheat seedlings with nutrient solution containing 0.6 mM Cd.
	*t*Zck	Irrigating wheat seedlings with nutrient solution containing 0.6 mM Cd + (grain soaking + 2 foliar sprays using 0.05 mM *t*Zck).
	Sim	Irrigating wheat seedlings with nutrient solution containing 0.6 mM Cd + (grain soaking + 2 foliar sprays using 0.5 mM Sim).
	*t*Zck + Sim	Irrigating wheat seedlings with nutrient solution containing 0.6 mM Cd + (grain soaking + 2 foliar sprays using 0.05 mM *t*Zck + 0.5 mM Sim).

Stm = Stimulator, Sim = Silymarin, tZck = *Trans*-zeatin-type cytokinin and Cd = Cadmium.

## Data Availability

Not applicable.

## Supplementary Materials

The following supporting information can be downloaded at: https://www.mdpi.com/article/10.3390/biology11081173/s1, Table S1. The composition of the nutrient solution used for watering wheat plants.
