# Supplementary material for: Exploring the Potential Enhancing Effects of Trans-Zeatin and Silymarin on the Productivity and Antioxidant Defense Capacity of Cadmium-Stressed Wheat
Source: Biology (Basel). 2022 Aug 4;11(8):1173. doi: 10.3390/biology11081173 (PMC9404800; doi:10.3390/biology11081173)
Supplement: Supplementary file 1 [file biology-11-01173-s001.zip › biology-1783575-supplementary.pdf]

Table S1. The composition of the nutrient solution used for watering wheat plants.

Chemical substance	Chemical formula	Amount (μM)
Calcium nitrate	$Ca(NO_3)_2$	2000
Potassium sulfate	K_2SO_4	700
Magnesium sulfate	$MgSO_4$	500
Monopotassium phosphate	KH_2PO_4	100
Potassium chloride	KCl	100
Boric acid	H_3BO_3	1
Manganese sulfate	$MnSO_4$	1
Copper sulfate	$CuSO_4$	0.25
Ammonium molybdate	$(NH_4)_6Mo_7O_{24}$	0.01
Fe–ethylenediaminetetraacetic acid (EDTA)	$(OOCCH_2)_2NCH_2CH_2NCCH_2COO)_2$ $FeNa \cdot xH_2O$	100
